# Immunostimulated Arginase II Expression in Intestinal Epithelial Cells Reduces Nitric Oxide Production and Apoptosis

**DOI:** 10.3389/fcell.2017.00015

**Published:** 2017-03-01

**Authors:** Maria M. Talavera, Sushma Nuthakki, Hongmei Cui, Yi Jin, Yusen Liu, Leif D. Nelin

**Affiliations:** ^1^Center for Perinatal Research, The Research Institute at Nationwide Children's HospitalColumbus, OH, USA; ^2^Department of Pediatrics, The Ohio State UniversityColumbus, OH, USA; ^3^Department of Pediatrics, Baylor College of Medicine, Texas Children's HospitalHouston, TX, USA

**Keywords:** inducible nitric oxide synthase, arginase, inflammation, necrotizing enterocolitis

## Abstract

Increased production of nitric oxide (NO) and subsequent local cytotoxicity to mucosal epithelial cells has been proposed as a putative mechanism involved in the development of necrotizing enterocolitis (NEC). Intestinal epithelial cells (IECs) metabolize L-arginine to either nitric oxide (NO) by NO synthase (NOS) or to L-ornithine and urea by arginase. L-ornithine is the first step in polyamine synthesis important for cell proliferation, while NO production can lead to apoptosis. We hypothesized that in IECs immunostimulation increases both NOS and arginase expression, and that arginase activity mitigates NO production and apoptosis. Rat intestinal epithelial cells (rIEC-6) were immunostimulated by either incubation with lipopolysaccharide (LPS) alone for 24 h or by incubation with conditioned media (CM) for 24 h. CM was obtained from RAW 264.7 cells (a macrophage cell line) treated with LPS (*E. coli* 0127:B8; 1 μg/ml) for 4 h. The rIEC-6 stimulated with LPS or with CM had significantly higher levels of inducible NOS (iNOS) protein, NO production, and arginase II protein than did the control cells. Direct LPS stimulation of rIEC-6 produced a less robust increase in iNOS expression and NO (represented as nitrite percent of control) than did CM stimulation. Inhibition of arginase using N^ω^ hydroxyl-L-arginine (NOHA) further increased stimulated NO production in rIEC-6. Viable cell numbers were significantly lower in CM stimulated cells after 24 h than in controls, and inhibition of arginase activity with NOHA resulted in a further significant decrease in viable cell numbers. We conclude that immunostimulated arginase expression of rIEC-6 cells tempers cytokine-induced iNOS-derived NO production and apoptosis.

## Introduction

Necrotizing enterocolitis (NEC) is the most common gastrointestinal emergency in preterm infants and a leading cause of neonatal morbidity and mortality (Lin and Stoll, 2006; Neu and Walker, 2011). The incidence among very low birth weight (VLBW) infants has remained constant at 5–7% for the past 30 years (Horbar et al., 2012). Mortality from NEC varies, depending on the amount of bowel involvement and the presence of comorbidities, with reports of up to 50% mortality in patients requiring surgery for NEC (Lin and Stoll, 2006). The pathogenesis of NEC remains unclear; however, it is likely the result of a multifactorial process in a susceptible host. Of particular interest is the role of inflammation-induced vascular dysfunction and epithelial cytotoxicity in the development of NEC (MacKendrick et al., 1993; Ford et al., 1997; Nowicki et al., 2005).

Nitric oxide (NO) is the principal inhibitory neurotransmitter in the gut, endothelial-derived NO is involved in the local regulation of mucosal blood flow and inflammatory-derived NO is involved in the loss of mucosal integrity (Stark and Szurszewski, 1992; Alican and Kubes, 1996). Increased production of NO and subsequent local cytotoxicity to mucosal epithelial cells has been proposed as one of the putative mechanisms in NEC development (Ford et al., 1997; Nadler et al., 2000; Chokshi et al., 2008). NO is synthesized from L-arginine by NO synthase (NOS), of which there are three isoforms inducible NOS (iNOS), endothelial NOS (eNOS) and neuronal NOS (nNOS) (Moncada, 1997). Of the three isoforms of NOS described, iNOS is not constitutively expressed, but induced at high levels during inflammation resulting in relatively high levels of NO production (Di Lorenzo et al., 1995; Chokshi et al., 2008). The role of iNOS-derived NO in the pathogenesis of NEC was first described by Ford and colleagues who demonstrated elevated iNOS expression in resected human NEC tissue compared to non-NEC control tissue (Ford et al., 1997). In experimental models of NEC, the inhibition of iNOS has been found to attenuate inflammatory intestinal injury (Ciftci et al., 2004; Giannone et al., 2006; Cintra et al., 2008).

L-arginine can be metabolized by arginase, of which there are two isoforms, arginase I, and arginase II. Arginase I has been described as the liver isoform, although it is found in many cell types throughout the body, while arginase II has been described as the inducible form of arginase and found in the kidney and small intestine (Badurdeen et al., 2015). Arginase converts L-arginine to L-ornithine, the first step in proline and polyamine synthesis, which are necessary for cell proliferation, collagen synthesis, and tissue regeneration. Alternatively, L-arginine metabolized by iNOS generates relatively high amounts of NO which can lead to cytotoxicity and apoptosis (Munder, 2009). We have previously found in endothelial cells and in macrophages that arginase and NOS compete for their common substrate, such that inhibiting arginase increased NO production (Chicoine et al., 2004; Jin et al., 2015). Therefore, we hypothesized that in intestinal epithelial cells inflammation would increase both NOS and arginase II protein expression, and that the inflammation-induced arginase activity acts to mitigate iNOS-derived NO production and thereby mitigates apoptosis. We used two different models of inflammation in these studies to induce iNOS and arginase. The first was direct treatment with lipopolysaccharide (LPS) to these cells as previously described (Talavera et al., 2015) and the second was to incubate the intestinal epithelial cells in conditioned media (CM) obtained from macrophages treated with LPS for 4 h. The LPS treatment gives insight into the direct effect of LPS on intestinal epithelial cells, while the CM, which would contain a mixture of cytokines and chemokines, provides insight into what may occur in the intestinal mileu during inflammation.

## Materials and methods

### Cell culture

Rat intestinal epithelial cells, rIEC-6, were purchased from the American Type Culture Collection (ATCC, Manassas, VA). rIEC-6 cells (studied in *passages* 15–20) are well-described, immortalized, immature, non-transformed rat small intestinal epithelial cells (Quaroni et al., 1979). Cells were grown in high glucose Dulbecco's Modified Eagle's Medium (DMEM, Mediatech, Manassas, VA) supplemented with 10% fetal bovine serum (FBS, Hyclone, Salt Lake City, UT), 100 U/ml penicillin, 100 μg/ml streptomycin, and 0.1 U/ml of recombinant human insulin. Cells were maintained in 100 mm tissue culture plates at 37°C in a humidified atmosphere with 5% CO_2_ in ambient air (21% O_2_). Cells were grown to a confluent monolayer prior to experimentation on 60 mm or 6-well plates (Thermo Fisher Scientific, Waltham, MA). Depending on the experiment, some wells were treated with LPS (*E. coli* 0127:B8; Sigma Chemicals) to a final media concentration of 100 μg/ml for 24 h. Other wells were treated with conditioned media (CM) for 24 h. The conditioned media was obtained by incubating a macrophage cell line (RAW 264.7 cells; American Type Culture Collection, Manassas, VA) with 1 μg/ml LPS (*E. coli* 0127:B8) for 4 h. The media was then harvested and placed on rIEC-6 cells for the indicated experiments as the immunostimulant.

### Nitrite assay

rIEC-6 cell media was collected in 1.5 ml tubes after treatment. The samples were assayed in duplicate for nitrite (${\text{NO}}_{2}^{-}$) using a chemiluminescence nitric oxide analyzer (model 280i; Sievers Instruments, Boulder, CO) as previously described (Nelin et al., 2001; Chicoine et al., 2004). Briefly, 50 μl of sample were placed in a reaction chamber containing a mixture of sodium iodide (NaI) in glacial acetic acid to reduce ${\text{NO}}_{2}^{-}$ to NO. The NO gas was carried into the NO analyzer by a constant flow of Helium gas. The analyzer was calibrated with a NaNO_2_ standard curve.

### Urea assay

The samples of medium were assayed in triplicate for urea concentration colorimetrically as previously described (Chicoine et al., 2004). Briefly, 100 μl of sample was added to 1.5 ml of chromogenic reagent (5 mg thiosemicarbazide, 250 mg diacetyl monoxime, 37.5 mg FeCl_3_ in 150 ml 25% (vol/vol) H_2_SO_4_, 20% (vol/vol) H_3_PO_4_). The mixtures were cooled to room temperatures and the absorbance (530 nm) was determined and compared with a urea standard curve.

### RNA isolation

RNA was isolated from rIEC-6 cells, as previously described (Talavera et al., 2015). Briefly, 0.7 ml of TRIzol Reagent (Invitrogen, Carlsbad, CA) was added to cells and incubated for 5 min at room temperature. Cells were scraped and the mixture collected in 1.5 ml centrifuge tubes. Chloroform (0.1 ml) was added, the tubes shaken for 15 s and incubated at 30°C for 3 min. The mixture was then centrifuged at 12,000 × g for 15 min at 4°C and the supernant collected. Isopropyl alcohol (0.25 ml) was added, the mixture was incubated at 30°C for 10 min and then centrifuged at 7,500 × g for 5 min at 4°C. The supernatant is then discarded, the pellet washed with 75% ethanol and centrifuged at 7,500 × g for 5 min at 4°C. The supernatant is again discarded, the pellet partially dried, dissolved in RNase-free water, and stored at −80°C.

### Quantitative real-time PCR

qPCR was performed as previously described (Talavera et al., 2015). Briefly, 4 μg of total RNA was pretreated with RQ1 RNase-free DNase (Promega) by incubating at 37°C for 30 min in a total volume of 10 μl. This reaction was terminated with the addition of RQ1 DNase stop solution. The reaction was then incubated at 65°C for 10 min to inactivate the DNase. The post-treated total RNA then underwent reverse transcription in a total volume of 40 μl containing 2.5 μM dT_16_ (Applied Biosystems, Foster City, CA), 20 units AMV-RT, 1 mM dNTP, 1x AMV RT buffer (Promega), and RNase-free water. The samples were incubated in a PCR-iCycler (Bio-Rad, Hercules, CA) at 42°C for 60 min, 95°C for 5 min, and stored at −20°C. Quantitative real-time PCR was performed with the Chromo 4 Real-time PCR Detection System (Bio-Rad), using qPCR SYBR Green Master-mix (Thermo Scientific). PCR reactions were performed for 40 cycles using the following parameters: 95°C for 15 s, 55°C for 30 s, 72°C for 30 s. The melting curves were manually verified for the presence of a single product. Arginase II was amplified using the forward primer (5′ AGAGAAGGCGGACACATTGCCTAT 3′) and the reverse primer (5′ TGTCGTGAAAGGTGCCAGAGTACA 3′). iNOS was amplified using the forward primer (5′ TGTAGCCGCTGTGTGTCACAGAAGT 3′) and the reverse primer (5′ AGCAAAGGCACAGAACTGAGGGTA 3′).18S was amplified using the forward primer (5′ CCAGAGCGAAAGCATTTGCCA 3′) and the reverse primer (5′ TCGGCATCGTTTATGGTCGGAACT 3′). For each reaction, negative controls containing reaction mixture and primers without cDNA were performed to verify that primers and reaction mixtures were free of template contamination. Relative arginase II and iNOS transcripts were normalized to 18S expression using ΔΔ CT method (Livak and Schmittgen, 2001).

### Protein isolation

Protein was isolated from rIEC-6 cell lysate as previously described (Jin et al., 2010; Talavera et al., 2015). Briefly, rIEC-6 cells were washed with ice-cold phosphate-buffered saline (PBS), and 50–100 μl of lysis solution (0.2M NaOH, 0.2% SDS) was added to each plate or each well of a six-well plate. Thirty minutes before use the following protease inhibitors were added to each ml of lysis solution: 1 μl aprotinin [10 mg/ml in double distilled (dd) H_2_O], l μl leupeptin (10 mg/ml in ddH_2_O), and 1 μl of phenylmethylsulfonyl fluoride (34.8 mg/ml methanol). The rIEC-6 cells were scraped, collected into sterile centrifuge tubes, and placed on ice for 30 min. The cell lysates were centrifuged at 12,000 × g for 15 min. The supernatant was stored at −80°C. Total protein concentration was determined by the Bradford method (Bradford, 1976) using a commercially available assay kit (Bio-Rad, Hercules, CA).

### Western blot

Cell lysate was assayed for protein levels of 3-nitro-tyrosine (3-NT), proliferating nuclear antigen (PCNA), iNOS, arginase I, arginase II, and cleaved-caspase-3 using immunoblot analyses as previously described (Chicoine et al., 2004; Talavera et al., 2015). Briefly, aliquots of cell lysate were diluted 1:1 using SDS sample buffer, heated to 80°C for 15 min, and then separated using SDS-polyacrylamide gel electrophoresis. The proteins were then transferred to polyvinylidene difluoride membranes and blocked overnight in TBS with 0.1% Tween (TBS-T) containing 5% nonfat dried milk. The membranes were then incubated with primary antibody overnight; 3-nitrotyrosine (3-NT) (1:750; Sigma Aldrich, St. Louis, MO), PCNA (1:5000; Sigma Aldrich), arginase I (1:500; Santa Cruz Biotechnology, Inc., Santa Cruz, CA), arginase II (1:500, Santa Cruz Biotechnology), cleaved caspase-3 (1:1,000, Cell Signaling Technology, Beverly, MA) or iNOS (1:1,000, BD BioSciences, San Jose, CA). The following day, membranes were washed three times with TBS-T, then incubated with horseradish peroxidase-conjugate goat anti-rabbit IgG (1:10,000, Bio-Rad) for 1 h, washed again three times with TBS-T. The protein bands were visualized using enhanced chemiluminescence (ECL plus reagent; Amersham Pharmacia Biotech, Piscataway, NJ) and quantified using densitometry (Sigma Gel, Jandel Scientific, San Rafael, CA). To control for protein loading, the blots were stripped using a stripping buffer containing 62.5 mM Tris HCl (pH 6.8), 2% SDS, and 100 mM β-mercaptoethanol, and the blots were reprobed for β-actin (1:10,000; Abcam, Cambridge, MA) as described above. Data are shown as the mean of the density of the protein of interest normalized to the density of β-actin.

### Proliferation assay

To determine cell proliferation, rIEC-6 cells were seeded in six-well plates at a density of 1 × 10^5^ cells/well. The cells were incubated in regular DMEM or in CM for 24 h and viable cells counted using trypan blue exclusion. To determine the effects of arginase inhibition on rIEC-6 proliferation following immunostimulation, cells were incubated in CM with either vehicle or 100 μM N^ω^-Hydroxy-nor-L-arginine (NOHA) added for 24 h and viable cells counted using trypan blue exclusion. In a separate set of proliferation experiments, using the same methodology as above, the effect of NOS inhibition on cell proliferation was determined by adding either vehicle or N^ω^-nitro-L-arginine methyl ester (L-NAME) to CM, incubating the cells for 24 h, and then counting the viable cells using trypan blue exclusion.

### Statistical analysis

Values are expressed as means ± SEM. The groups were compared using a *t*-test when 2 groups were compared or a one-way analysis of variance (ANOVA) or two-way ANOVA when more than two groups were compared. For the ANOVA a Student-Newman-Keuls *post-hoc* test was used to identify significant differences. Differences were considered significant when *p* < 0.05 (Prism; GraphPad Software, San Diego, CA).

## Results

### LPS-induced nitric oxide production in rIEC-6 cells

**Figure 1:**
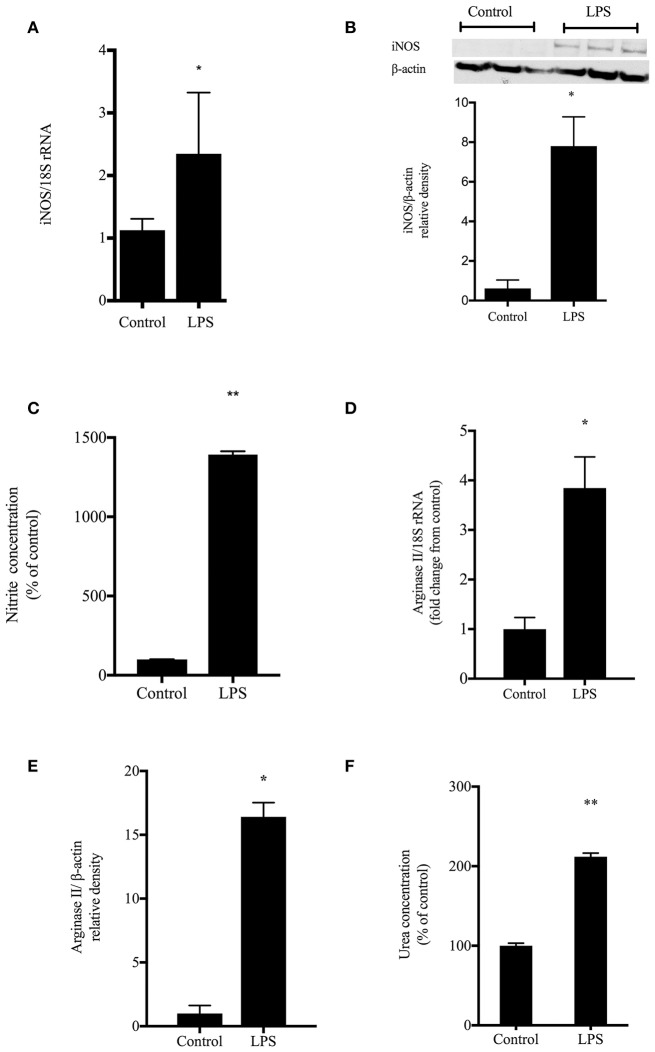
**LPS induced NO and urea production**. rIEC-6 cells were treated with LPS or vehicle (control) for 24 h. **(A)** iNOS mRNA levels detected by qRT-PCR normalized to 18srRNA levels (*n* = 6 for each group). **(B)** Cell lysates were harvested and iNOS protein levels determined by Western blot. The densitometry data (*n* = 3 for each group) normalized to β-actin are shown relative to control. **(C)** Nitrites were analyzed by chemiluminescence for media harvested after a 24 h incubation with vehicle (control) or LPS (*n* = 9 for each group). Nitrite levels were measured in media and represented as a percent of control. **(D)** Cell lysates were examined for arginase II mRNA levels using qPCR normalized to 18s rRNA (*n* = 6 for each group). Densitometry results are shown as fold change from control (where control levels = 1). **(E)**. Cell lysates were examined for arginase II protein levels by Western blot (*n* = 4 for each group). The densitometry data normalized to β-actin are shown relative to control. **(F)**. Urea production was determined by colorimetric assay after harvesting medium from cells treated with vehicle (control) or LPS (*n* = 6 for each group). Urea levels were measured in media and are represented as a percent of control urea values. ^*^LPS different from control, *p* < 0.05, ^**^LPS different from control, *p* < 0.001.

To determine the effects of LPS on nitric oxide and urea production in enterocytes, cells were grown to ~ 80% confluence, treated with LPS (*E. coli* 0127:B8) or left untreated (control) for 24 h. The cells were harvested for RNA and analyzed by qRT-PCR. There was increased iNOS mRNA levels (*p* < 0.05) from LPS stimulated cells as compared with unstimulated IECs (Figure 1A). Cell lysates were collected and evaluated by Western blot for iNOS protein levels. The LPS-treated cells showed ~7-fold greater iNOS protein expression than control (*p* < 0.001) (Figure 1B). In a similar set of experiments, the cell media was examined for production of nitrites as a marker of NO production. LPS-treated cells had substantially greater (*p* < 0.001) NO concentration (represented as percent control) than did control cells (Figure 1C).

To examine LPS effects on arginase, cell lysates were assayed for arginase I and arginase II mRNA and protein levels. There was no significant effect of LPS on arginase I mRNA or protein levels in these rIEC-6 cells (data not shown). However, LPS-treated cells had substantially greater levels of arginase II mRNA (Figure 1D) and arginase II protein (Figure 1E) than did control cells. The cell media was also assayed for urea production. The LPS-treated rIEC-6 cells significantly greater (*p* < 0.001) urea concentration (represented at percent of control) that did vehicle-treated cells (Figure 1F).

### LPS-induced nitrite production is enhanced by arginase inhibition

**Figure 2:**
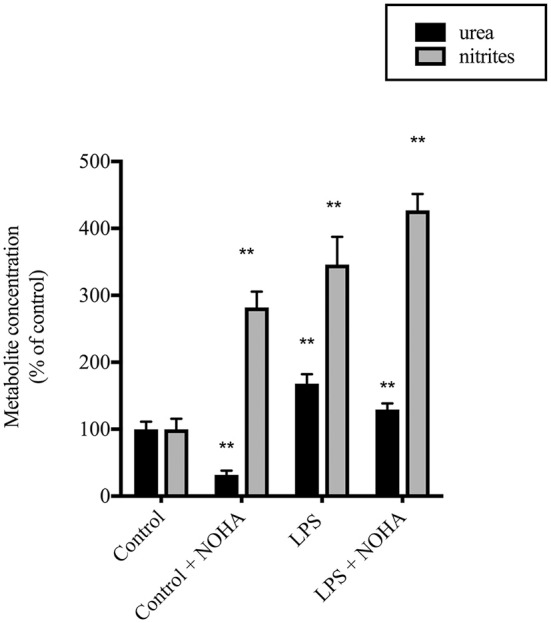
**LPS-induced NO production was augmented when urea production was inhibited**. rIEC-6 cells were incubated for 24 h in media containing LPS and either vehicle or 100 μM N^ω^-Hydroxy-nor-L-arginine acetate (NOHA). Urea (black bars) was measured using a colorimetric assay and the results are presented as percent of control (i.e., no LPS) urea values. Nitrites (gray bars) were measured in the media and are presented as a percent of control nitrite values. ^**^LPS different from control; ^**^LPS+NOHA different from control + NOHA, *p* < 0.001; multiple *t*-test and 2-way ANOVA.

To determine the role of arginase activity in LPS-induced NO production in rIEC-6 cells we utilized the putative arginase inhibitor NOHA. Cells were either not treated or treated with LPS and the LPS-treated cells were also treated with vehicle or NOHA (100 μM). The media was collected 24 h later and assayed for nitrites and urea. LPS-treated cells had significantly greater urea production than control cells and NOHA treatment attenuated the LPS-induced increase in urea production (Figure 2). LPS-treated cells had substantially greater NO production than did control cells and treatment with NOHA augmented LPS-induced NO production (Figure 2). These findings are consistent with the concept that arginase acts to limit iNOS-derived NO production in LPS stimulated rIEC-6 cells.

### Arginase inhibition results in greater cytotoxicity and decreased proliferation in LPS-treated rIEC-6 cells

**Figure 3:**
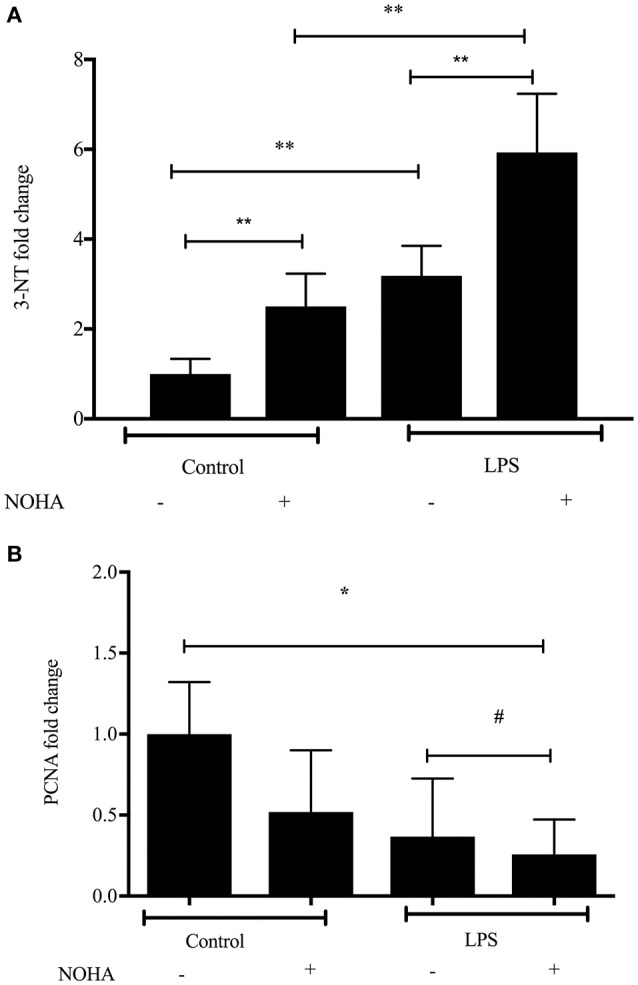
**Arginase inhibition resulted in increased 3-nitrotyrosine and decreased proliferation in LPS-treated rIEC-6 cells**. rIEC-6 cells were treated with LPS or untreated (control), and either with the arginase inhibitor (NOHA) or its vehicle for 24 h. Cell lysate was examined for 3-NT, PCNA or β-actin by Western blot. **(A)**. Densitometry data for 3-NT normalized to β-actin and presented as fold change from control (*n* = 6 for each group); ^**^LPS+ NOHA-treated different from control + NOHA; LPS + NOHA different from LPS alone; LPS different from control; Control + NOHA different from control, *p* < 0.001. **(B)**. Densitometry data for PCNA normalized to β-actin and presented as fold change from control (*n* = 9 for each group); #LPS different from LPS+NOHA control, *p* < 0.05. ^*^LPS+NOHA different from control, *p* < 0.001.

To examine the effects of arginase inhibition on NO-mediated cytotoxicity in LPS-treated rIEC-6 cells, we performed Western blot analyses for 3-nitrotyrosine—(3-NT) protein expression. 3-NT is a marker for peroxynitrite formation, which is formed in the presence of NO and superoxide during conditions of oxidative stress. LPS-treatment resulted in a significant increase in 3-NT level and treatment with LPS + NOHA resulted in greater 3-NT levels than in LPS-treated cells (Figure 3A). Inhibtion of arginase in control cells also led to a significant production of 3-NT but not significantly more than arginase inhibition following LPS stimulation (*p* < 0.001) (Figure 3A).

We also examined protein levels of proliferating cell nuclear antigen (PCNA), a marker of cell proliferation in each experimental group, hypothesizing that if NO production were greater in the LPS treated cells that PCNA levels would be lower and that arginase inhibition would further lower PCNA protein levels. We found a significantly lower level of PCNA protein in LPS-treated cells than in control cells (Figure 3B). Furthermore, when arginase was inhibited in the LPS-treated cells the levels of PCNA were lowest (Figure 3B). These results indicate that arginase inhibition in LPS-treated enterocytes lead greater nitrosative damage and decreased cell proliferation.

### CM increases iNOS and arginase II protein levels

**Figure 4:**
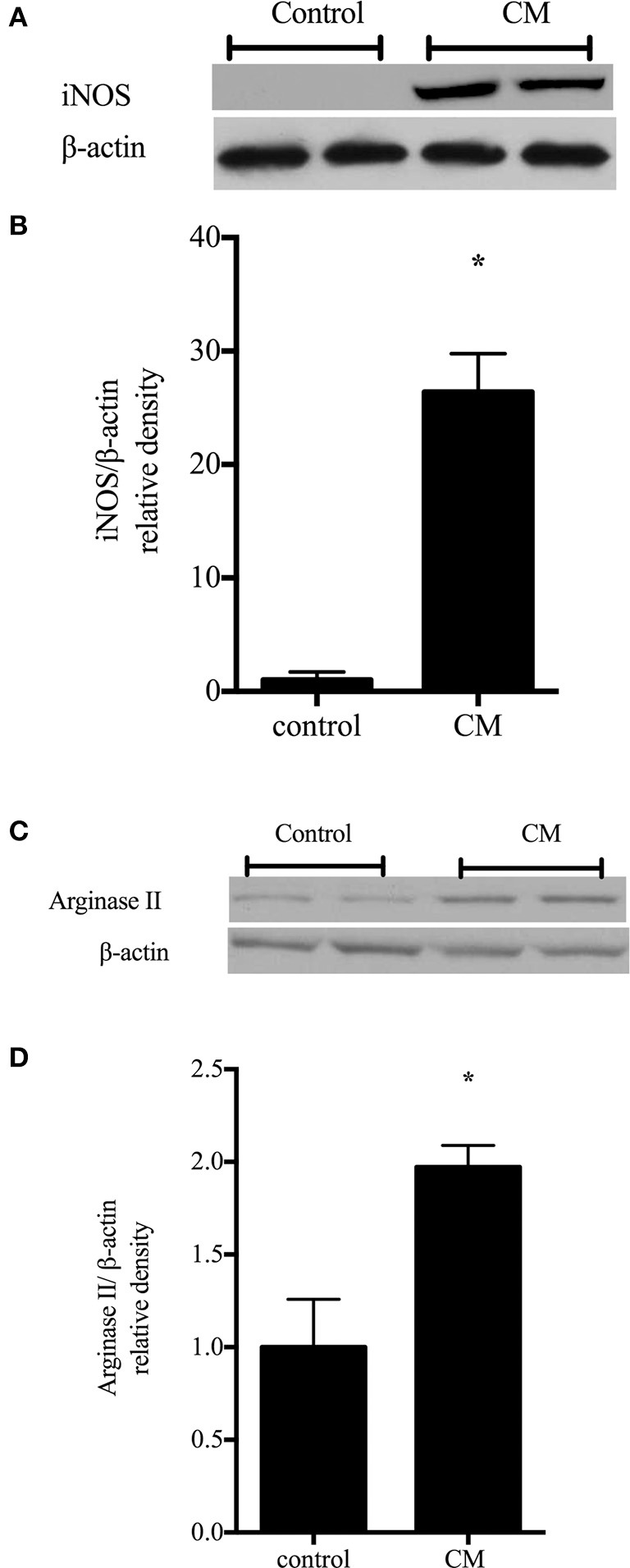
**Conditioned media up-regulates iNOS and arginase II protein levels**. rIEC-6 cells were exposed to conditioned media (CM) for 24 h. CM media (DMEM with FBS) was collected from RAW 264.7 cells stimulated with 1 μg/ml LPS for 4 h. **(A)** Cell lysates (*n* = 6 for each group) were examined for protein levels of iNOS by western blotting. **(B)** The densitometry data normalized to β-actin are shown relative to control. **(C)** Cell lysates (*n* = 6 for each group) were examined for protein levels of arginase II by western blotting. **(D)** The densitometry data normalized to β-actin are shown relative to control. ^*^CM different from control, *p* < 0.005.

To examine the effect of another form of immunostimulation on iNOS and arginase II protein levels rIEC-6 cells were treated with either CM or regular media (control) for 24 h. Cell lysates were examined for iNOS and arginase II protein levels by Western blot analysis. Following CM stimulation, iNOS protein levels were substantially higher than in control cells (Figure 4A). The fold increase in iNOS protein levels with CM relative to control were substantially greater than the fold increase in iNOS protein levels with LPS stimulation alone. Similarly, in cells incubated in CM the arginase II protein levels were significantly higher than in the control cells (Figure 4B). Interestingly, the fold change in arginase II levels relative to control caused by CM was similar to that seen with LPS.

### Treatment with CM increased apoptosis in rIEC-6 cells

**Figure 5:**
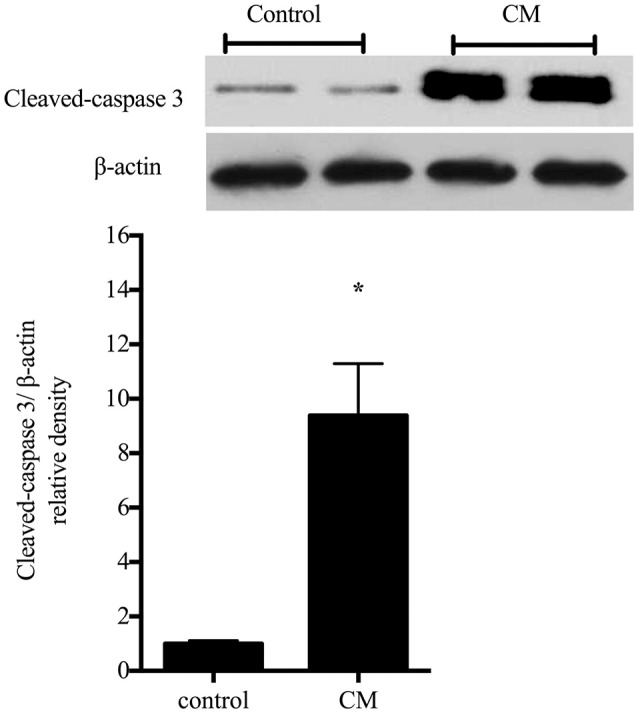
**Condtioned media increased apoptosis in rIEC-6 cells**. Cells were exposed to conditioned media (CM) for 24 h. CM media (DMEM and FBS) was collected from RAW 264.7 cells stimulated with 1 μg/ml LPS for 4 h. Cell lysates were examined for levels of cleaved caspase-3 by western blotting. Typical western blots for cleaved caspase-3 and β-actin. The bar graph shows the densitometry data for cleaved caspase 3 normalized to β-actin (*n* = 6 for each group) are shown relative to control (i.e., control = 1). ^*^CM different from control, *P* < 0.005.

To begin to examine the effects of iNOS induction on cell viability, the protein levels of cleaved caspase-3 were determined in rIEC-6 cells following incubation in CM. The cleaved caspase-3 levels were nearly 10-fold greater (*p* < 0.0001) in rIEC-6 cells incubated in CM compared to control cells (Figure 5). This data suggest that the CM-induced increase in iNOS protein levels in rIEC-6 cells was associated with the greater apoptosis.

### Inhibition of arginase results in greater NO production and fewer viable cells

**Figure 6:**
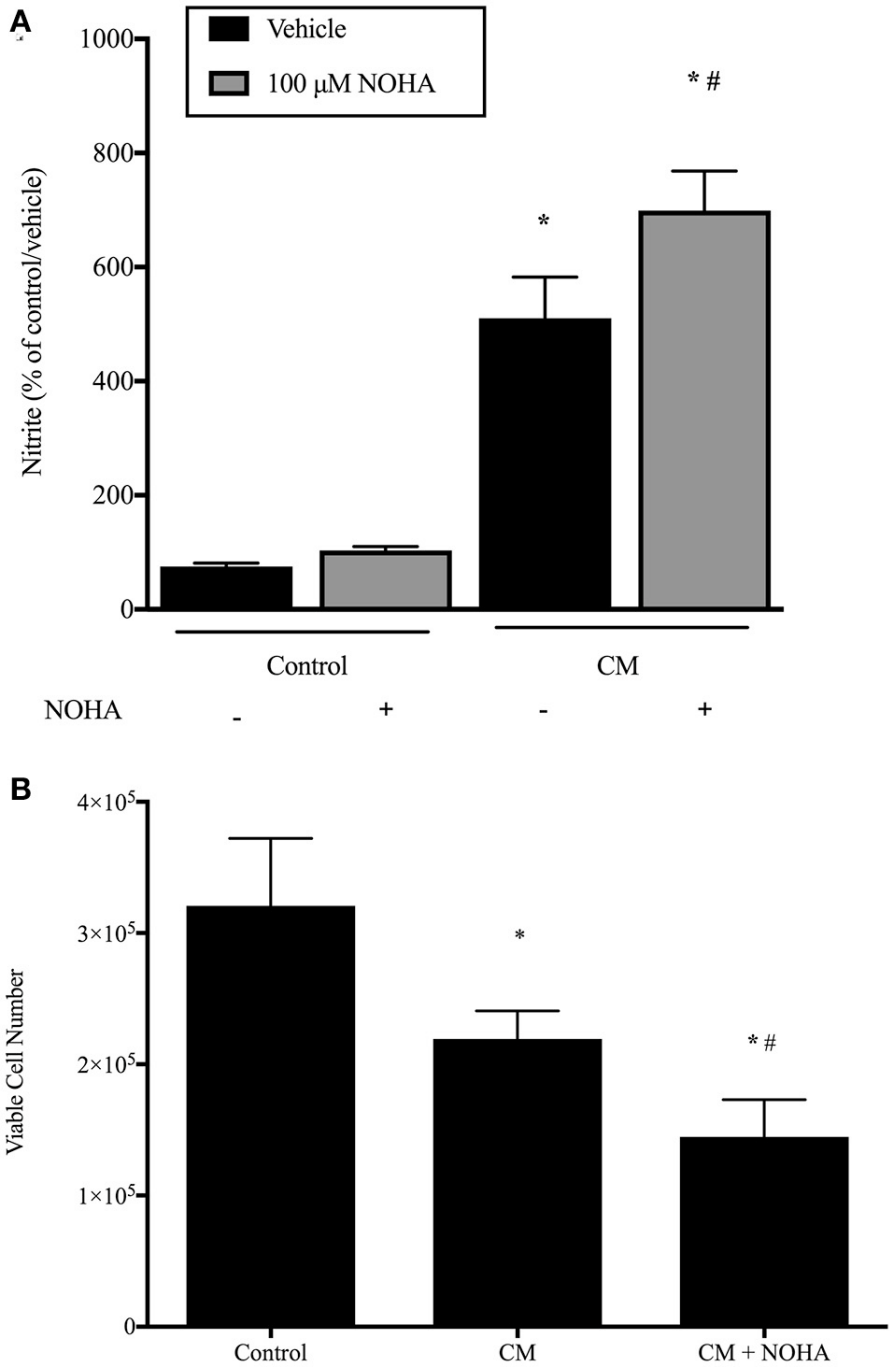
**Conditioned media results in more NO production and fewer viable cells and inhibition of arginase augments this response**. rIEC-6 cells were incubated in either regular media (control) or conditioned media (CM), and with either vehicle or 100 μM NOHA added for 24 h. **(A)**. Media was harvested for determination of NO production by assaying nitrites using chemiluminescence. Conditioned media resulted in a substantially greater NO production in vehicle treated cells (black bars) than did regular media (control). Inhibiting arginase with NOHA (gray bars) augmented the CM-induced production of NO. ^*^CM different from control, *p* < 0.001. # NOHA different from vehicle, *p* < 0.005. **(B)**. Equal numbers of rIEC-6 cells were seeded in each well of 6 well plates and treated as above. After 24 h viable cell numbers were determined using trypan blue exclusion. ^*^CM different from control, *p* < 0.05. #CM + NOHA different from CM, *p* < 0.05.

To determine the effects of CM and arginase inhibition on rIEC-6 NO production, rIEC-6 cells were grown to 80% confluence and then incubated in CM or regular media with either vehicle or 100 μM NOHA added for 24 h. Nitrite levels were measured in the media. Similar to our findings with LPS alone, treatment with CM resulted in a substantially greater nitrite levels than in control cells (Figure 6A). Inhibiting arginase with NOHA in the CM-treated cells resulted in substantially greater nitrite levels than in the CM and vehicle treated cells (Figure 6A).

To determine the effect of CM on viable cell number, rIEC-6 cells were seeded at a density of 1 × 10^5^ cells/well in a six-well plate. These cells were then treated with regular media (control), CM or CM with 100 μM NOHA for 24 h and viable cell numbers determined using trypan blue exclusion. Treatment of rIEC-6 cells with CM resulted in significantly fewer viable cells than in control rIEC-6 cells (Figure 6B). Addition of NOHA to the CM resulted in significantly fewer viable cells than in the cells incubated in CM and vehicle (Figure 6B). These findings support a role for arginase in attenuating iNOS generated NO production to preserve rIEC-6 cell viability following inflammatory stimuli.

### Inhibiting arginase augments apoptosis, while inhibiting NOS attenuates apoptosis

**Figure 7:**
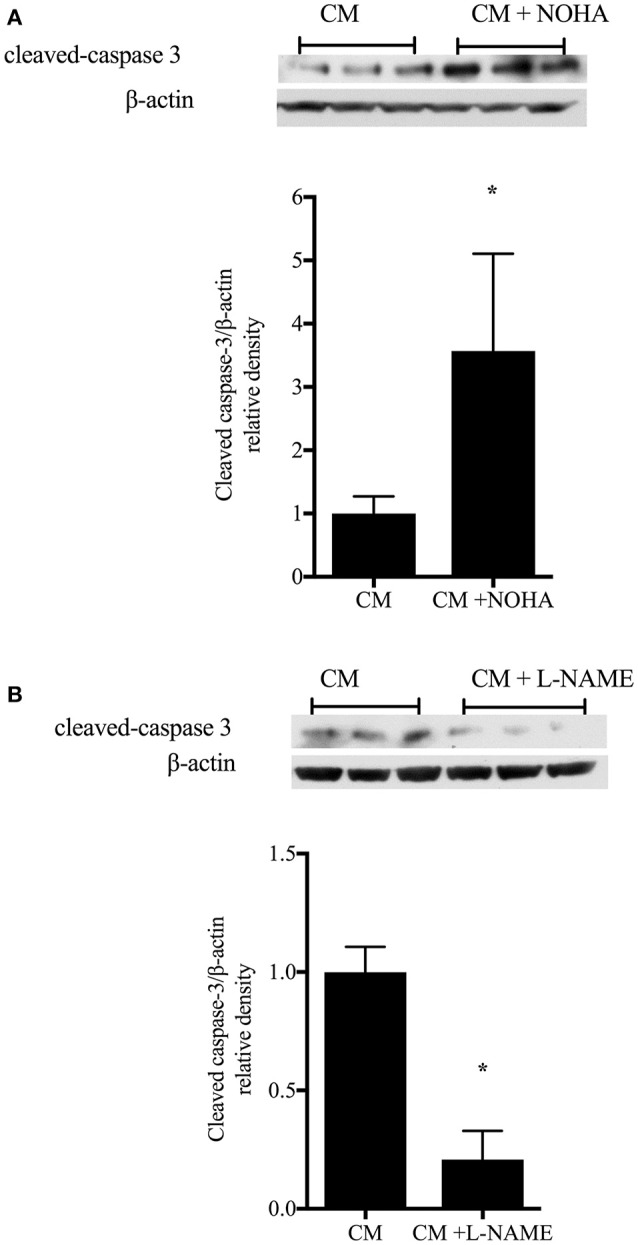
**Inhibition of arginase augments, while inhibition of iNOS attenuates, enterocyte apoptosis**. rIEC-6 cells were grown ~80% confluence then incubated in either conditioned media (CM) alone or CM and the arginase inhibitor, NOHA **(A)**, or CM alone or CM + the iNOS inhibitor, L-NAME **(B)**, for 24 h. Cell lysates were collected and protein harvested to determine cleaved caspase-3 and β-actin protein levels. **(A)**. Representative Western blots for cleaved caspase-3 and β-actin in CM and CM + NOHA-treated cells (*n* = 9 each group). The bar graph is the densitometry data for cleaved caspase-3 normalized to β-actin and shown relative to CM alone. ^*^p < 0.05 CM + NOHA different than CM alone. **(B)**. Representative Western blots for cleaved caspase-3 and β-actin in CM and CM + L-NAME-treated cells (*n* = 9 for each group). The bar graph is the densitometry data of cleaved caspase-3 normalized to β-actin as shown relative to CM alone. ^*^p < 0.001 CM + L-NAME different than CM alone.

To determine the role of arginase inhibition on enterocyte apoptosis, rIEC-6 cells were grown to ~80% confluence then stimulated with either CM or CM + NOHA (100 μM) for 24 h. Cell lysate was collected and protein harvested. Cleaved caspase-3 protein levels were determined by Western blot analyses. We found significantly greater cleaved caspase- 3 protein levels in the rIEC-6 cells treated with CM + NOHA than in cell treated with CM alone (Figure 7A). To determine the role of immunostimulated NO production on enterocyte apoptosis, rIEC-6 were incubated in either CM or CM + L-NAME (100 μM) for 24 h. Cell lysates was collected and protein harvested. Cleaved-caspase-3 protein levels were determined by Western blot analyses. The CM + L-NAME treated cells had significantly lower (*p* < 0.001) cleaved caspase-3 protein levels than did cells incubated in CM alone (Figure 7B). These results suggest that during intestinal inflammatory conditions arginase expression in enterocytes may temper iNOS-mediated apoptosis.

## Discussion

The major findings of this study in rIEC-6 were that: (1) immunostimulation increased iNOS and arginase II protein levels, as well as NO and urea production; (2) inhibiting arginase resulted in greater immunostimulated NO production; (3) immunostimulation increased apoptosis; (4) inhibition of arginase resulted in further decreased viable cell number and increased apoptosis following immunostimulation; and (5) inhibition of NOS production attenuated apoptosis following immunostimulation. These findings support our hypothesis that immunostimulation of intestinal epithelial cells increased both iNOS and arginase II protein expression and that arginase activity mitigated iNOS-derived NO production and subsequent apoptosis.

In this study we used two methods of experimental immunostimulation of rIEC-6 cells: direct LPS stimulation and incubation with conditioned media from LPS-stimulated macrophage cell line. The conditioned media would be expected to contain biologically relevant levels of a wide-variety of chemokines and cytokines. Intestinal epithelial cells (IECs) are in nearly constant contact with bacteria and bacterial products (LPS) and during inflammatory states there can be significant influx of inflammatory cells including macrophages (De Plaen, 2013). IEC's express cytokine receptors and secrete cytokines in response to inflammatory stimuli (Jung et al., 1995; Reinecker and Podolsky, 1995). We have previously shown that rIEC-6 cells express both TNF-α and COX-2 following direct LPS stimulation (Talavera et al., 2015). In this study, we found that both direct LPS and cytokine-induced (CM) immunostimulation resulted in greater expression of both arginase II and iNOS in rIEC-6 cells. Our data supports the notion that enterocyte-derived iNOS is up-regulated during inflammatory states and is responsible for high levels of NO production (Grishin et al., 2016).

These high levels of NO production can exert detrimental effects on the gut barrier leading to increased permeability, increased bacterial translocation (Hackam, 2011), impaired mitochrondrial function (Erusalimsky and Moncada, 2007), and impaired epithelial restitution following injury (Cetin et al., 2007). We found that enterocyte-derived iNOS expression in response to both LPS and conditioned media conditions parallel elevated NO levels (Figures 1, 3) and cleaved caspase-3 (Figure 4) expression. Cytoplasmic NO readily and very rapidly reacts with superoxide to form peroxynitrite, which is cytotoxic to enterocytes by the formation of nitrotyrosine residues alterating protein enzymatic activity (Schopfer et al., 2003; Grishin et al., 2016). We have shown that direct LPS stimulation following arginase inhibition significantly increased 3-nitrotyrosine levels and decreased PCNA levels in rIEC-6 cells. We also demonstrated that in the presence of a small molecule arginase inhibitor, NOHA, iNOS-dependent NO production was increased in rIEC-6 cells, and that this increase in NO production resulted in fewer viable cells. Thus, our data supports a central role of iNOS in enterocyte apoptosis, which would be expected to lead to decreased barrier function *in vivo*.

The opposing biological effects of iNOS and arginase in response to inflammation are mainly due to the cytopathic effects of NO compared to the regenerative, proliferative effects of arginase on the cellular environment (Hibbs, 1991; Chokshi et al., 2008). Others have shown how specific inflammatory ligands will determine a predominant iNOS or arginase expression. For example, in murine macrophages Th2 cytokines (IL-4 and IL-10) are potent inducers of arginase, whereas Th1 cytokine -INF-γ is a potent inducer of iNOS (Modolell et al., 1995). Similarly, in mouse peritoneal exudate cells, the growth factor, TGF-β, attenuated INF-γ-induced increased iNOS activity resulting in increased arginase activity (Shearer et al., 1997). In this study, we demonstrated that direct LPS stimulation of rIEC-6 cells resulted in arginase II induction and increased urea production. Concurrently, direct LPS stimulation also induced iNOS activity and increased NO production. It is unclear if iNOS and arginase II induction following either LPS or CM stimulation results from similar signaling cascades or if different signaling cascades are involved. In RAW cells, we recently reported that extracellular-signal regulated kinase (ERK) was involved in arginase II induction but that p38 was necessary for iNOS induction following LPS stimulation (Jin et al., 2015). Further studies will be needed in enterocytes to determine if similar cellular mechanisms are involved in immune-mediated iNOS and arginase II induction.

The dynamic interplay between iNOS and arginase activities has been reported in other cell types including vascular endothelium, macrophages and gastrointestinal epithelium (Eckmann et al., 2000; Berkowitz et al., 2003; Chicoine et al., 2004; Miki et al., 2009; Jin et al., 2015). In this report we studied the relative roles of immunostimulated iNOS and arginase II in intestinal epithelial cell NO production. We demonstrated that treatment with conditioned media (CM) induced a significantly greater iNOS expression compared to arginase II in rIEC-6 cells. We also demonstrated that cytokine-induced nitrite production was increased by arginase inhibition in rIEC-6 cells. These findings are consistent with a previous study in LPS/TNF-α-stimulated endothelial cells, wherein inhibition of arginase resulted in an increase in NO production (Chicoine et al., 2004). Our lab has also demonstrated that when LPS-induced arginase II expression in RAW 264.7 cells was prevented using a siRNA against arginase II, iNOS-derived NO production was substantial enhanced (Jin et al., 2015). These findings suggest that following immunostimulation in various cell types including enterocytes, arginase and iNOS compete for their common substrate, L-arginine, such that decreasing arginase activity leads to an increase in NO production. Thus, arginase activity is involved in cellular regulation of inflammatory NO production in intestinal epithelial cells.

Enhanced apoptosis in intestinal epithelial cells is a hallmark of intestinal barrier dysfunction, which is thought to be an important pathogenic feature for NEC in preterm infants (De Plaen, 2013). The increased enterocyte apoptosis observed in NEC has been shown to correlate with an increase in iNOS activity and 3-NT staining (Nadler et al., 2000). In this study, we demonstrated that inhibition of iNOS-induced NO production attenuated apoptosis in rIEC-6 cells, as evidenced by substantially lower levels of cleaved caspase-3 in cells treated with a NOS inhibitor, L-NAME. While augmenting iNOS-derived NO production by arginase inhibition resulted in significantly greater levels of cleaved caspase-3. These findings highlight the cytotoxic effects of iNOS-derived NO in rIEC cells and the role of arginase activation in attenuating inflammation-induced NO-mediated apoptosis.

In conclusion, we demonstrated that inflammation-induced NO production in rIEC-6 is iNOS mediated and leads to cytotoxicity and decreased viable cell numbers. We found that inflammation-induced arginase activity in enterocytes mitigates the effects of iNOS-derived NO production by attenuating NO production and the resultant apoptosis. These findings suggest that inhibition of iNOS and/or augmentation arginase II activities in enterocytes may be viable therapeutic strategies to prevent epithelial cell apoptosis and loss of barrier function. It is important to remember though that some level of NO is needed to maintain mucosal homeostasis, vascular tone, and oxidative stress, while relatively high levels of NO, as seen with iNOS induction, lead to apoptosis. Our findings suggest a potential therapeutic target would be augmented arginase activity in inflamed intestinal epithelial cells as a means to prevent disease progression and facilitate early proliferation and repair. We further speculate that neonates with lower degrees of arginase induction, perhaps due to genetic mutations in their arginase II genes, may be more prone to develop NEC.

## Author contributions

MT, SN, YL, and LN contributed to the conceptualization, data analysis and manuscript preparation. SN, HC, and YJ contributed to conducting studies and data collection. All authors contributed to the conceptualization and organization of the manuscript as well as final manuscript approval.

### Conflict of interest statement

The authors declare that the research was conducted in the absence of any commercial or financial relationships that could be construed as a potential conflict of interest.

## References

[B1] Alican I. Kubes P. (1996). A critical role for nitric oxide in intestinal barrier function and dysfunction. Am. J. Physiol. 270, G225–G237. 8779963 10.1152/ajpgi.1996.270.2.G225

[B2] Badurdeen S. Mulongo M. Berkley J. A. (2015). Arginine depletion increases susceptibility to serious infections in preterm newborns. Pediatr. Res. 77, 290–297. 10.1038/pr.2014.177 25360828 PMC4335378

[B3] Berkowitz D. E. White R. Li D. Minhas K. M. Cernetich A. Kim S.. (2003). Arginase reciprocally regulates nitric oxide synthase activity and contributes to endothelial dysfunction in aging blood vessels. Circulation 108, 2000–2006. 10.1161/01.CIR.0000092948.04444.C7 14517171

[B4] Bradford M. M. (1976). A rapid and sensitive method for the quantitation of microgram quantities of protein utilizing the principle of protein-dye binding. Anal Biochem. 72, 248–254. 10.1016/0003-2697(76)90527-3 942051

[B5] Cetin S. Leaphart C. L. Li J. Ischenko I. Hayman M. Upperman J.. (2007). Nitric oxide inhibits enterocyte migration through activation of RhoA-GTPase in a SHP-2-dependent manner. Am. J. Physiol. Gastrointest. Liver Physiol. 292, G1347–G1358. 10.1152/ajpgi.00375.2006 17272518

[B6] Chicoine L. G. Paffett M. L. Young T. L. Nelin L. D. (2004). Arginase inhibition increases nitric oxide production in bovine pulmonary arterial endothelial cells. Am. J. Physiol. Lung Cell. Mol. Physiol. 287, L60–L68. 10.1152/ajplung.00194.2003 14977627

[B7] Chokshi N. K. Guner Y. S. Hunter C. J. Upperman J. S. Grishin A. Ford H. R. (2008). The role of nitric oxide in intestinal epithelial injury and restitution in neonatal necrotizing enterocolitis. Semin. Perinatol. 32, 92–99. 10.1053/j.semperi.2008.01.002 18346532 PMC2390779

[B8] Ciftci I. Dilsiz A. Aktan T. M. Gurbilek M. Duman S. (2004). Effects of nitric oxide synthase inhibition on intestinal damage in rats with experimental necrotizing enterocolitis. Eur. J. Pediatr. Surg. 14, 398–403. 10.1055/s-2004-821105 15630641

[B9] Cintra A. E. Martins J. L. Patricio F. R. Higa E. M. Montero E. F. (2008). Nitric oxide levels in the intestines of mice submitted to ischemia and reperfusion: L-arginine effects. Transplant. Proc. 40, 830–835. 10.1016/j.transproceed.2008.02.044 18455030

[B10] De Plaen I. G. (2013). Inflammatory signaling in necrotizing enterocolitis. Clin. Perinatol. 40, 109–124. 10.1016/j.clp.2012.12.008 23415267 PMC3579498

[B11] Di Lorenzo M. Bass J. Krantis A. (1995). Use of L-arginine in the treatment of experimental necrotizing enterocolitis. J. Pediatr. Surg. 30, 235–240; discussion 240–231. 10.1016/0022-3468(95)90567-7 7537808

[B12] Eckmann L. Laurent F. Langford T. D. Hetsko M. L. Smith J. R. Kagnoff M. F.. (2000). Nitric oxide production by human intestinal epithelial cells and competition for arginine as potential determinants of host defense against the lumen-dwelling pathogen *Giardia lamblia*. J. Immunol. 164, 1478–1487. 10.4049/jimmunol.164.3.1478 10640765

[B13] Erusalimsky J. D. Moncada S. (2007). Nitric oxide and mitochondrial signaling: from physiology to pathophysiology. Arterioscler. Thromb. Vasc. Biol. 27, 2524–2531. 10.1161/ATVBAHA.107.151167 17885213

[B14] Ford H. Watkins S. Reblock K. Rowe M. (1997). The role of inflammatory cytokines and nitric oxide in the pathogenesis of necrotizing enterocolitis. J. Pediatr. Surg. 32, 275–282. 10.1016/S0022-3468(97)90194-9 9044137

[B15] Giannone P. J. Schanbacher B. L. Bauer J. A. Reber K. M. (2006). Effects of prenatal lipopolysaccharide exposure on epithelial development and function in newborn rat intestine. J. Pediatr. Gastroenterol. Nutr. 43, 284–290. 10.1097/01.mpg.0000232572.56397.d6 16954948

[B16] Grishin A. Bowling J. Bell B. Wang J. Ford H. R. (2016). Roles of nitric oxide and intestinal microbiota in the pathogenesis of necrotizing enterocolitis. J. Pediatr. Surg. 52, 13–17. 10.1016/j.jpedsurg.2015.10.006. PMC4894644 26577908

[B17] Hackam D. J. (2011). Danger at the doorstep: regulation of bacterial translocation across the intestinal barrier by nitric oxide. Crit. Care Med. 39, 2189–2190. 10.1097/CCM.0b013e31822661ad 21849832

[B18] Hibbs J. B. Jr. (1991). Synthesis of nitric oxide from L-arginine: a recently discovered pathway induced by cytokines with antitumour and antimicrobial activity. Res. Immunol. 142, 565–569; discussion 596–568. 10.1016/0923-2494(91)90103-P 1812549

[B19] Horbar J. D. Carpenter J. H. Badger G. J. Kenny M. J. Soll R. F. Morrow K. A.. (2012). Mortality and neonatal morbidity among infants 501 to 1500 grams from 2000 to 2009. Pediatrics 129, 1019–1026. 10.1542/peds.2011-3028 22614775

[B20] Jin Y. Calvert T. J. Chen B. Chicoine L. G. Joshi M. Bauer J. A.. (2010). Mice deficient in Mkp-1 develop more severe pulmonary hypertension and greater lung protein levels of arginase in response to chronic hypoxia. Am. J. Physiol. Heart Circ. Physiol. 298, H1518–H1528. 10.1152/ajpheart.00813.2009 20173047 PMC2867445

[B21] Jin Y. Liu Y. Nelin L. D. (2015). Extracellular signal-regulated kinase mediates expression of arginase II but not inducible nitric-oxide synthase in lipopolysaccharide-stimulated macrophages. J. Biol. Chem. 290, 2099–2111. 10.1074/jbc.M114.599985 25451938 PMC4303663

[B22] Jung H. C. Eckmann L. Yang S. K. Panja A. Fierer J. Morzycka-Wroblewska E.. (1995). A distinct array of proinflammatory cytokines is expressed in human colon epithelial cells in response to bacterial invasion. J. Clin. Invest. 95, 55–65. 10.1172/JCI117676 7814646 PMC295369

[B23] Lin P. W. Stoll B. J. (2006). Necrotising enterocolitis. Lancet 368, 1271–1283. 10.1016/S0140-6736(06)69525-1 17027734

[B24] Livak K. J. Schmittgen T. D. (2001). Analysis of relative gene expression data using real-time quantitative PCR and the 2^−ΔΔC_T_^ Method. Methods 25, 402–408. 10.1006/meth.2001.1262 11846609

[B25] MacKendrick W. Caplan M. Hsueh W. (1993). Endogenous nitric oxide protects against platelet-activating factor-induced bowel injury in the rat. Pediatr. Res. 34, 222–228. 10.1203/00006450-199308000-00025 8233729

[B26] Miki K. Kumar A. Yang R. Killeen M. E. Delude R. L. (2009). Extracellular activation of arginase-1 decreases enterocyte inducible nitric oxide synthase activity during systemic inflammation. Am. J. physiol. Gastrointest. Liver Physiol. 297, G840–G848. 10.1152/ajpgi.90716.2008 19713467 PMC2763806

[B27] Modolell M. Corraliza I. M. Link F. Soler G. Eichmann K. (1995). Reciprocal regulation of the nitric oxide synthase/arginase balance in mouse bone marrow-derived macrophages by TH1 and TH2 cytokines. Eur. J. Immunol. 25, 1101–1104. 10.1002/eji.1830250436 7537672

[B28] Moncada S. (1997). Nitric oxide in the vasculature: physiology and pathophysiology. Ann. N.Y. Acad. Sci. 811, 60–67; discussion 67–69. 10.1111/j.1749-6632.1997.tb51989.x 9186585

[B29] Munder M. (2009). Arginase: an emerging key player in the mammalian immune system. Br. J. Pharmacol. 158, 638–651. 10.1111/j.1476-5381.2009.00291.x 19764983 PMC2765586

[B30] Nadler E. P. Dickinson E. Knisely A. Zhang X. R. Boyle P. Beer-Stolz D.. (2000). Expression of inducible nitric oxide synthase and interleukin-12 in experimental necrotizing enterocolitis. J. Surg. Res. 92, 71–77. 10.1006/jsre.2000.5877 10864485

[B31] Nelin L. D. Nash H. E. Chicoine L. G. (2001). Cytokine treatment increases arginine metabolism and uptake in bovine pulmonary arterial endothelial cells. Am. J. physiol. Lung Cell. Mol. Physiol. 281, L1232–L1239. Available online at: http://ajplung.physiology.org/content/281/5/L1232.full.pdf+html 11597915 10.1152/ajplung.2001.281.5.L1232

[B32] Neu J. Walker W. A. (2011). Necrotizing enterocolitis. New Engl. J. med. 364, 255–264. 10.1056/NEJMra1005408 21247316 PMC3628622

[B33] Nowicki P. T. Dunaway D. J. Nankervis C. A. Giannone P. J. Reber K. M. Hammond S. B.. (2005). Endothelin-1 in human intestine resected for necrotizing enterocolitis. J. Pediatr. 146, 805–810. 10.1016/j.jpeds.2005.01.046 15973323

[B34] Quaroni A. Wands J. Trelstad R. L. Isselbacher K. J. (1979). Epithelioid cell cultures from rat small intestine. Characterization by morphologic and immunologic criteria. J. Cell Biol. 80, 248–265. 10.1083/jcb.80.2.248 88453 PMC2110349

[B35] Reinecker H. C. Podolsky D. K. (1995). Human intestinal epithelial cells express functional cytokine receptors sharing the common gamma c chain of the interleukin 2 receptor. Proc. Natl. Acad. Sci. U.S.A. 92, 8353–8357. 10.1073/pnas.92.18.8353 7667294 PMC41155

[B36] Schopfer F. J. Baker P. R. Freeman B. A. (2003). NO-dependent protein nitration: a cell signaling event or an oxidative inflammatory response? Trends Biochem. Sci. 28, 646–654. 10.1016/j.tibs.2003.10.006 14659696

[B37] Shearer J. D. Richards J. R. Mills C. D. Caldwell M. D. (1997). Differential regulation of macrophage arginine metabolism: a proposed role in wound healing. Am. J. Physiol. 272, E181–E190. 9124321 10.1152/ajpendo.1997.272.2.E181

[B38] Stark M. E. Szurszewski J. H. (1992). Role of nitric oxide in gastrointestinal and hepatic function and disease. Gastroenterology 103, 1928–1949. 10.1016/0016-5085(92)91454-C 1333429

[B39] Talavera M. M. Kralik N. Jin Y. Chen B. Liu Y. Nelin L. D. (2015). Mitogen-activated protein kinase phosphatase-1 prevents lipopolysaccharide-induced apoptosis in immature rat intestinal epithelial cells. Pediatr. Res. 78, 128–136. 10.1038/pr.2015.88 25950450 PMC7500060

